# Suizide im deutschen Strafvollzug: Häufigkeit, Risikofaktoren und Prävention

**DOI:** 10.1007/s00103-021-03460-4

**Published:** 2021-12-29

**Authors:** Annette Opitz-Welke, Norbert Konrad

**Affiliations:** 1 Institut für Forensische Psychiatrie, Charité – Universitätsmedizin Berlin, Campus Virchow-Klinikum, Außenstandort (Haus 10), Oranienburger Str. 285, 13437 Berlin, Deutschland; 2 Justizvollzugskrankenhaus, Abteilung für Psychiatrie und Psychotherapie, Justizvollzugsanstalt (JVA) Plötzensee, Berlin, Deutschland

**Keywords:** Gefängnis, Untersuchungsgefangene, Screening, Suizidprävention, Psychiatrische Erkrankung, Prison, Prisoners on remand, Screening, Suicide prevention, Psychiatric disorder

## Abstract

Gefangene haben ein hohes Suizidrisiko und die höchsten Suizidraten sind bei Untersuchungsgefangenen zu verzeichnen. Suizid ist die häufigste singuläre Todesursache in deutschen Gefängnissen. In diesem narrativen Übersichtsbeitrag werden zunächst die Gefängnispopulation und ihre allgemeine Gesundheitsversorgung beschrieben, wobei insbesondere auf psychiatrische und Substanzkonsumstörungen eingegangen wird. Der Hauptteil widmet sich der Prävalenz, den Ursachen und Risikofaktoren von Gefängnissuizid. Maßnahmen zur Suizidprävention werden dargestellt.

Der Anteil von Männern unter Gefangenen in Deutschland ist mit 94 % (2020) wie in allen Teilen der Welt sehr hoch. Die meisten Gefangenen sind jungen oder mittleren Alters. Die durchschnittlichen jährlichen Suizidraten bei Männern und Frauen in deutschen Gefängnissen entsprechen mit 105,8/100.000 bzw. 54,7/100.000 denen der meisten Länder in der Europäischen Union (Vollerhebung 2000–2011). Die Suizidraten bei männlichen deutschen Gefangenen sind in den Jahren 2000–2013 unabhängig vom Alter kontinuierlich zurückgegangen. Bei weiblichen Gefangenen stiegen sie dagegen an, wobei die Ursachen hierfür nicht bekannt sind. Es gibt Hinweise darauf, dass psychiatrische Erkrankungen nicht erkannt worden waren.

Wichtige suizidpräventive Maßnahmen sind die Unterbringung in Gemeinschaft und die Vermeidung von Isolation, beispielsweise durch das Angebot von Arbeit. Zudem stehen validierte deutschsprachige Screeninginstrumente zur Verfügung, um ein Suizidrisiko frühzeitig zu erkennen. Für eine wirksame Gefängnissuizidprävention bedarf es der Identifikation von Hochrisikopersonen, des Angebots geeigneter suizidpräventiver Maßnahmen sowie der Entwicklung teambezogener Maßnahmen beim Gefängnispersonal.

## Abstract (en)

Prisoners have a high risk of dying by suicide and the highest suicide rates are recorded among prisoners on remand. A death by suicide is the most common single cause of death in German correctional institutions. This narrative overview first describes the prison population and its general healthcare needs by paying particular attention to psychiatric and substance use disorders. The main section attends to the prevalence, causes, and risk factors of prison suicide. Suicide prevention measures are presented.

As in all parts of the world, the proportion of young men among prisoners in Germany is very high (94% in 2020). Most prisoners are young or middle-aged. The average annual suicide rates among men (105.8/100,000) and women (54.7/100,000) in German prisons are similar to those of most countries in the European Union (full census 2000–2011). Suicide rates among male German prisoners declined continuously from 2000–2013, regardless of age. In contrast, they increased among female prisoners; the reasons for this are not known. Evidence suggests that psychiatric disorders have not been identified.

Important suicide prevention measures include shared accommodation and avoidance of solitary confinement, for example by offering work. In addition, validated German-language screening instruments are available to detect suicide risk at an early stage. For effective prison suicide prevention, identifying high-risk individuals, offering appropriate suicide prevention interventions, and developing team-based interventions among prison staff are required.

## Hintergrund

Gefangene in Deutschland haben im Vergleich zur Allgemeinbevölkerung ein vielfach erhöhtes Suizidrisiko und Untersuchungsgefangene sind innerhalb dieser Gruppe besonders stark gefährdet. Internationale Vergleiche der Höhe von Suizidraten bei Gefangenen und der entsprechenden Allgemeinbevölkerung zeigten keinen Zusammenhang. Die Beobachtung, dass Personen, die von Strafverfolgung betroffen waren, auch nach Abschluss des Verfahrens bzw. nach Verbüßung einer Freiheitsstrafe ein erhöhtes Suizidrisiko haben, legt einerseits das Vorhandensein individueller Risikofaktoren nahe. Auf der anderen Seite spricht besonders die hohe Suizidrate der Untersuchungsgefangenen, die in allen untersuchten Gefängnispopulationen festgestellt wurde, für das Einwirken institutionell-situativer Einflüsse auf das Suizidrisiko im Gefängnis [1–4].

Suizide im Gefängnis zeigen besondere Charakteristika, welche die Notwendigkeit spezifischer Maßnahmen in diesem Lebensumfeld begründen. Zum vertieften Verständnis der Besonderheiten von Suizidhandlungen im Gefängnis sind Kenntnisse über die spezifischen Lebensbedingungen von Gefangenen und über die Wirksamkeit präventiver und therapeutischer Maßnahmen innerhalb der Gefängnismauern (intramurale Wirksamkeit) notwendig.

Im Folgenden werden zum besseren Verständnis zunächst die demografischen und gesundheitsbezogenen Besonderheiten von Gefängnispopulationen herausgearbeitet und die Struktur der Gesundheitsversorgung im Gefängnis erläutert. Dann wird der aktuelle wissenschaftliche Kenntnisstand zu Prävalenz, Genese und Prävention von Suiziden im Gefängnis unter Berücksichtigung der Gruppen mit spezifischen Bedürfnissen dargestellt und abschließend ausgeführt, welche Maßnahmen zur Verbesserung der Versorgung suizidaler Gefangener sinnvoll erscheinen und wie diese Maßnahmen in der aktuellen Versorgungslandschaft umgesetzt werden könnten.

## Die Gefängnispopulation: Charakteristika und Gesundheitsversorgung

Weltweit sind aktuell rund 11 Mio. Menschen inhaftiert, wobei sich in den Vereinigten Staaten von Amerika, im Verhältnis zur Bevölkerungszahl, die meisten Menschen in Gefangenschaft befinden (700/100.000 Einwohner; [5]). In der Europäischen Union sind aktuell ungefähr 500.000 Menschen inhaftiert und die Gesamtgefangenenrate lag nach der Strafstatistik des Europarates, die auf Daten aus den offiziellen nationalen Statistiken beruht, 2018 im Median bei 102,5 Gefangene pro 100.000 Einwohner und war zwischen 2016 und 2018 um insgesamt 6,6 % gesunken. Deutschland zählt im europäischen Vergleich mit einer Gefangenenrate von 77,5/100.000 Einwohner zu den Ländern mit einer leicht unterdurchschnittlichen Gefangenenrate [5, 6]. Unabhängig von der Inhaftierungsrate ist bei Gefangenen in allen Teilen der Welt der Anteil von Männern jungen und mittleren Alters sehr hoch. So waren im Juni 2020 in Deutschland nach Angaben des statistischen Bundesamtes 94 % der Gefangenen männlich und nur 4 % älter als 60 Jahre [7].

Nach den Ergebnissen von Untersuchungen zur Prävalenz psychiatrischer Störungen bei Gefangenen in Deutschland liegt bei bis zu 8 % der Gefangenen eine Schizophrenie vor, 20–44 % leiden an einer Alkoholabhängigkeit und bei bis zu 40 % kann eine depressive Störung diagnostiziert werden [8]. Eine systematische Übersichtsarbeit und Metaanalyse zur Nikotinabhängigkeit im Gefängnis auf der Grundlage von Daten aus 36 Nationen aus den Jahren 2012–2016 ermittelte im Vergleich zu der jeweiligen Allgemeinbevölkerung eine 1,04- bis 62,60-fach höhere Prävalenz [9].

Ob Inhaftierung selbst die Entstehung psychiatrischer Störungen auslösen kann, wird seit Jahrzehnten diskutiert. Allerdings ist die Hypothese einer haftspezifischen psychischen Erkrankung (Haftpsychose) mittlerweile widerlegt [10]. Nach klinischer Erfahrung liegt den meisten in Haft festgestellten psychotischen Syndromen eine Schizophrenie zugrunde. Seltener handelt es sich um Personen mit einer drogeninduzierten psychotischen Störung.

Bei Gefangenen in Deutschland ist die Prävalenz der Abhängigkeit von Opioiden sehr hoch. Nach Ergebnissen einer durch das Robert Koch-Institut durchgeführten repräsentativen Querschnittuntersuchung gab ein knappes Drittel der Befragten an, schon einmal intravenös Drogen konsumiert zu haben, und von den Heroinkonsumenten gaben 22,7 % der Befragten an, auch innerhalb der Justizvollzugsanstalten zu konsumieren [11]. In einer aktuellen Untersuchung zur Häufigkeit von Opioidabhängigkeit und Substitutionsbehandlung bei Berliner Gefangenen waren 16 % der Gefangenen opioidabhängig, wovon sich 42 % während ihres Gefängnisaufenthaltes in Substitutionsbehandlung befanden [12]. Bezogen auf den Konsum anderer illegaler Substanzen und Alkohol liegen keine systematischen Untersuchungen zur Prävalenz bei Gefängnisinsassen vor. Nach klinischer Erfahrung variiert das Substanzkonsummuster innerhalb der Justizvollzugsanstalten in Abhängigkeit von den lokalen Angeboten außerhalb, wobei grundsätzlich alle illegalen Drogen und Alkohol auf dem anstaltsinternen Schwarzmarkt erhältlich sind [8].

Die Gesundheitsversorgung der Gefangenen ist in Deutschland stärker primärärztlich orientiert als die ambulanten Versorgungsangebote außerhalb der Justizvollzugsanstalten und es gibt – abgesehen von der Möglichkeit, sich im Rahmen der Untersuchungshaft auf eigene Kosten innerhalb der Justizvollzugsanstalten behandeln zu lassen – keine freie Wahl bezüglich Ärztin oder Arzt. Die ersten Ansprechpersonen bei gesundheitlichen Problemen sind die Anstaltsärztinnen und -ärzte, die meist der Fachrichtung Allgemeinmedizin oder auch der inneren Medizin (mit hausärztlicher Tätigkeit) angehören. Bei Bedarf werden andere Fachrichtungen konsiliarisch hinzugezogen. Die stationäre Versorgung wird durch Justizvollzugskrankenhäuser und Krankenabteilungen sichergestellt, wobei trotz der Häufigkeit psychiatrischer Erkrankungen nur in einem Teil der Bundesländer die Justizvollzugskrankenhäuser auch über eine psychiatrische Abteilung verfügen. Der Umfang der angebotenen medizinischen Leistungen orientiert sich an dem sogenannten Äquivalenzprinzip, das bedeutet, dass das Angebot dem durch die gesetzliche Krankenversicherung vorgehaltenen entsprechen sollte. Dabei sind einige Angebote innerhalb der Justizvollzugsanstalten schwieriger umzusetzen als andere. So haben Gefangene mit Substanzkonsumstörungen in der Regel während einer Inhaftierung nur sehr eingeschränkten Zugang zu Angeboten der ambulanten Suchthilfe, was insbesondere die Überleitung in ambulante Suchthilfeangebote nach Haftentlassung erschwert.

Eine Inhaftierung wird von dem meisten Gefangenen als Niederlage und negative Lebenserfahrung empfunden. Trotzdem konnte bisher nicht nachgewiesen werden, dass eine lange Inhaftierung in Deutschland psychische Schäden verursacht [13]. Möglicherweise wirken einige Faktoren der Entstehung psychiatrischer Störungsbilder eher entgegen. So werden Gefangene im Vergleich zur Allgemeinbevölkerung medizinisch bei Bedarf auch aufsuchend betreut, Alkohol ist in deutschen Gefängnissen nur in sehr geringem Umfang verfügbar und die Lebensführung ist generell reguliert, beispielsweise durch den nächtlichen Einschluss und das regelmäßige Angebot von Mahlzeiten. Da aber Untersuchungen fehlen, die die langfristige Entwicklung der Gesundheit von Gefangenen mit der entsprechenden Allgemeinbevölkerung vergleichen, ist ein möglicher ungünstiger Einfluss der Inhaftierung auf die Gesundheit nicht ausreichend erforscht.

## Prävalenz von Gefängnissuizid

Nach den Ergebnissen der Analyse einer Vollerhebung aller im Zeitraum 2000–2011 in Deutschland an Suizid verstorbenen Gefangenen war die Suizidrate der männlichen Gefangenen 5,5-mal höher als die der männlichen Allgemeinbevölkerung im selben Zeitraum, bei weiblichen Gefangenen war die entsprechende Rate 8,7-mal höher [14]. Die durchschnittlichen jährlichen Gefängnissuizidraten von 105,8/100.000 (Männer) bzw. 54,7/100.000 (Frauen) entsprechen denen der meisten Länder der Europäischen Union [15]. Dabei war die Suizidrate der männlichen Untersuchungsgefangenen mit 291,5/100.000 fünfmal höher als die der Strafgefangenen und bei weiblichen mit 136,4/100.000 sogar sechsmal höher [14]. Unter Berücksichtigung der Tatsache, dass Suizide in deutschen Gefängnissen Ursache für ungefähr die Hälfte aller Todesfälle in diesem Zeitraum gewesen sind und dass in Deutschland kein erhöhtes Sterblichkeitsrisiko aufgrund anderer Ursachen, wie beispielsweise in Osteuropa mit dort erhöhter Prävalenz bei Strafgefangenen, besteht, ist davon auszugehen, dass Suizide in Deutschland in diesem Zeitraum die häufigste singuläre Todesursache im Gefängnis gewesen sind [14, 16].

Als Ergebnis der Analyse einer Vollerhebung aller an Suizid verstorbenen Gefangenen in Deutschland im Zeitraum 2000–2013 konnte festgestellt werden, dass 17,6 % der Verstorbenen 50 Jahre oder älter waren. Der Vergleich der Suizidraten von älteren Gefangenen (>50 Jahre) und jüngeren Gefangenen (<50 Jahre) zeigte, dass die Suizidrate bei den Älteren mit 204,9/100.000 im Untersuchungszeitraum fast doppelt so hoch war wie bei den Jüngeren mit 115,7/100.000. In Hinblick auf die Entwicklung der Gefängnissuizidraten von 2000 bis 2013 wurde bei den männlichen Gefangenen und auch in der Gruppe der älteren Gefangenen insgesamt ein rückläufiger Trend deutlich [17]. Die Suizidrate der weiblichen Gefangenen zeigte im Gegensatz dazu einen ansteigenden Trend, sodass weibliche Gefangene als Hochrisikogruppe für Gefängnissuizid einzustufen sind [18].

## Ursachen und Risikofaktoren

Insbesondere zu Beginn einer Inhaftierung und in der Untersuchungshaft entwickeln viele Gefangene reaktiv-depressive Störungsbilder. Einwirkende Belastungsfaktoren sind das hohe Maß an externer Kontrolle, stark eingeschränkte Kontaktmöglichkeiten zu Personen außerhalb der Justizvollzugsanstalten, Auseinandersetzung mit dem laufenden Strafverfahren und die Konfrontation mit der anstaltsinternen Subkultur. Anpassungsstörungen mit depressiver und gelegentlich auch paranoider Symptomatik treten besonders bei Untersuchungsgefangenen häufig auf und sind allgemein als Ausdruck der erhöhten psychosozialen Vulnerabilität von Gefangenen aufzufassen [19]. Einerseits kann Inhaftierung für ein Individuum in einer spezifischen Situation eine unlösbar erscheinende Konfliktsituation darstellen, in der, im Sinn des „Krisenmodells der Suizidalität“, eine Selbsttötungsabsicht als Reaktion oder Dekompensation einer eigentlich ansonsten ausreichend stabilen Persönlichkeit verstanden werden kann. Auf der anderen Seite leiden Gefangene im Vergleich zur Allgemeinbevölkerung häufiger unter psychiatrischen Erkrankungen und Substanzkonsumstörungen, die als Risikofaktoren für Suizidalität gut belegt und konzeptionell dem „Krankheitsmodell der Suizidalität“ zuzuordnen sind [20, 21].

Untersuchungen zu individuellen Suizidrisikofaktoren im Gefängnis konnten feststellen, dass Gefangene ein erhöhtes Suizidrisiko hatten, wenn die Diagnose einer psychiatrischen Erkrankung oder einer Substanzkonsumstörung vorlag. Auch eine bereits vorhandene positive Suizidanamnese ging mit einem erhöhten Suizidrisiko einher [22]. Wie in den meisten Ländern haben auch in Deutschland Männer ein höheres Risiko, im Gefängnis an Suizid zu versterben, als Frauen [4] und junge Gefangene sind im Vergleich zur entsprechenden Altersgruppe in der Allgemeinbevölkerung stärker suizidgefährdet [23]. Als weitere Risikofaktoren für den Tod durch Suizid im Gefängnis konnten identifiziert werden: der Status als Untersuchungshäftling, lediger Familienstand, Inhaftierung wegen Gewalt- oder Sexualstraftat oder wegen geringfügiger Delikte [4, 24–26]. Die Suizidrate war außerdem erhöht bei Unterbringung in überfüllten Gefängnissen, bei Einzelunterbringung [27], bei Obdachlosigkeit oder generell bei sozial isolierten oder traumatisierten Personen und bei denjenigen, die innerhalb der Justizvollzugsanstalten Opfer von Schikanen anderer Gefangener geworden waren (Bullying; [28–30]). Zudem erwiesen sich einige ethnische Gruppen als stärker suizidgefährdet. So waren beispielsweise in den Vereinigten Staaten von Amerika Gefangene kaukasischer Herkunft stärker suizidgefährdet als Gefangene afrikanischer oder lateinamerikanischer Herkunft. In Australien war das Risiko für Gefängnissuizid bei den Nachfahren indigener Australier im Vergleich zu Australiern anderer Abstammung erhöht. Als protektiver Faktor bzgl. Gefängnissuizid erwies sich eine tagesstrukturierende Beschäftigung [31, 32].

Die Vielzahl der identifizierten Risikofaktoren zeigt, dass die hohen Suizidraten im Gefängnis wahrscheinlich das Ergebnis einer Interaktion verschiedener Einflussgrößen sind, wobei Evidenz über das Zusammenwirken der einzelnen Faktoren fehlt [33]. Der Vergleich der Suizidrate von Inhaftierten mit den Suizidraten von Personen, die im psychiatrischen Maßregelvollzug untergebracht sind, ermöglicht eine Einschätzung der Einflussstärke institutioneller Suizidrisikofaktoren. Die Zuweisung erfolgt in beiden Systemen durch Dritte und gesellschaftliche Sicherheitsinteressen wirken sich auf die psychiatrische Arbeit aus [34]. In einer vergleichenden Untersuchung von Daten aus einer Befragung der psychiatrischen Maßregelvollzugskliniken aus den Jahren 2000–2004 mit Daten aus einer Vollerhebung aller Suizide von deutschen Gefangenen im selben Zeitraum zeigte sich lediglich ein geringer Unterschied in der Höhe der Suizidrate (Maßregelvollzugskliniken: 123/100.000, Justizvollzugsanstalten: 130/100.000; [35]). In der Gruppe der Gefangenen war der Anteil der Untersuchungsgefangenen signifikant höher und bei signifikant weniger Personen wurden zurückliegende Suizidversuche berichtet. In den Justizvollzugsanstalten war nur bei 7 % der an Suizid verstorbenen Gefangenen das Vorliegen einer psychiatrischen Störung bekannt. Es ist aber davon auszugehen, dass der Anteil psychiatrischer Erkrankungen in dieser Gruppe deutlich unterschätzt wurde, denn Untersuchungen zur Suizidepidemiologie kommen zu dem Ergebnis, dass allgemein bei 90–98 % aller an Suizid Verstorbenen eine psychiatrische Erkrankung vorgelegen hat [35]. Da die Maßregelvollzugskliniken auf die Behandlung psychiatrischer Erkrankungen ausgerichtet sind, erscheint nachvollziehbar, dass psychiatrische Störungsbilder in diesen Einrichtungen zuverlässiger erfasst wurden als in den Justizvollzugsanstalten. Außerdem sind Interventionen in den Justizvollzugsanstalten stärker auf den Umgang mit aggressiven Personen ausgerichtet und Personen, die sich aufgrund einer psychiatrischen Störung ruhig und zurückgezogen verhalten, wird in der Regel weniger Aufmerksamkeit zuteil [36]. Insbesondere Rückzug und gedrückte Stimmung fallen in Justizvollzugsanstalten mit langen Einschlusszeiten und daraus folgendem eingeschränkten Kontaktangebot wenig auf oder werden als angemessene Reaktion fehlverstanden, obwohl eigentlich eine depressive Entwicklung vorliegt. Für eine wirksame Suizidprävention ist deshalb auch eine bedarfsgerechte Gestaltung von Kontaktmöglichkeiten sinnvoll.

## Gruppen mit speziellen Bedürfnissen: weibliche und ältere Gefangene

Bei der Analyse von Risikofaktoren für Gefängnissuizid wurden weibliche Gefangene als besonders gefährdete Teilgruppe identifiziert [18]. Alle Gefangenen werden in Deutschland bei Aufnahme ärztlich untersucht und bei Bedarf an Fachärztinnen und Fachärzte überwiesen. Die Beobachtung, dass in der Gruppe der an Suizid verstorbenen weiblichen Gefangenen 3 Viertel vormals psychiatrisch untersucht worden waren, aber nur die Hälfte der an Suizid verstorbenen männlichen Gefangenen, könnte als indirekter Hinweis auf eine höhere Prävalenz psychiatrischer Störungen bei Frauen gewertet werden. Die bei Frauen im Vergleich signifikant häufiger vorliegenden Drogenentzugssyndrome lassen auf eine höhere Prävalenz von Substanzkonsumstörungen schließen. Für die Beantwortung der Frage, ob die psychiatrisch-psychotherapeutischen Angebote innerhalb der Justizvollzugsanstalten in Deutschland auf die Bedürfnisse der inhaftierten Frauen zugeschnitten sind, liegen keine Untersuchungen vor. Allerdings werden aufgrund der geringen Anzahl weiblicher Gefangener stationäre Behandlungsangebote häufig exklusiv für Männer vorgehalten.

In Berlin wurde in einer Untersuchung von 150 weiblichen Gefangenen bei insgesamt 62 % (95 % Konfidenzintervall (KI): 54–70) der Stichprobe das Vorliegen einer Substanzkonsumstörung festgestellt [37]. Bei somit großem Bedarf an suchtmedizinischen Angeboten gaben 66 % (95 % KI: 58–73) der Stichprobe an, bereits vor der Inhaftierung in psychiatrischer Behandlung gewesen zu sein. Hier zeigen sich also keine Hinweise darauf, dass diese Gruppe grundsätzlich durch Interventionen schwer zu erreichen gewesen wäre [38]. Möglicherweise wird durch die Geschlechtertrennung, die als eine strukturell bedingte institutionelle Einflussgröße aufzufassen ist, ein bedarfsgerechtes Behandlungsangebot für inhaftierte Frauen verhindert. Ähnliche Einflüsse des Geschlechts sind aus der geschlechtsbezogenen Gesundheitsforschung für die Behandlungsergebnisse bei anderen Krankheitsbildern bekannt [39].

Auch die Untersuchung der älteren an Suizid verstorbenen Gefangenen ist geeignet abzuschätzen, ob die Zugehörigkeit zu einer Minderheit Einfluss auf das Suizidrisiko haben könnte. In Deutschland sind 15 % der Gefängnisbevölkerung älter als 50 Jahre und 4 % älter als 60 Jahre. Ähnliche Zahlen sind aus anderen Industrienationen bekannt [17]. Obwohl zahlreiche internationale Untersuchungen eine erhöhte Belastung älterer Gefangener mit psychiatrischen Störungen, insbesondere depressiven Syndromen, berichten, ergaben entsprechende Analysen der Daten in Deutschland keine Hinweise auf eine erhöhte Häufigkeit psychiatrischer Symptome in dieser Gruppe [40].

Insbesondere die vielen älteren männlichen Gefangenen sind im Gegensatz zu weiblichen Gefangenen nicht durch das Gebot der Geschlechtertrennung von spezifischen Angeboten ausgeschlossen. Im Gegensatz zu der Gruppe der weiblichen Gefangenen werden sie innerhalb der Justizvollzugsanstalten in der Regel als potenziell benachteiligte Gruppe wahrgenommen. Diese Einschätzung ist durch die Tatsache bestimmt, dass das soziale Klima und die Lebensbedingungen innerhalb der Justizvollzugsanstalten verstärkt auf die Bedürfnisse jüngerer Männer ausgerichtet sind. Räumlich höher gelegene Hafträume und medizinische Behandlungseinheiten sind, insbesondere in den älteren Justizvollzugsanstalten, nicht immer barrierefrei zu erreichen, Transportwege und Beschäftigungsangebote nur für körperlich rüstige Erwachsene ausgelegt. Bei altersbedingter Funktionseinschränkung sind die älteren Gefangenen in der Regel auf die Unterstützung durch das medizinische Fachpersonal angewiesen, denn die Regelversorgung, die ebenfalls eher auf jüngere, körperlich leistungsfähige Erwachsene ausgerichtet ist, hält keine Ressourcen für zusätzliche Leistungen, wie beispielsweise Unterstützung in Aktivitäten des täglichen Lebens oder aufsuchende Versorgung, bereit.

Einige Untersuchungen belegen eine verbesserte Anpassungsfähigkeit älterer Gefangener im Vergleich zu den jüngeren; im Alltag der Justizvollzugsanstalten kann beobachtet werden, dass ältere Männer selten in Rivalitätskonflikte verwickelt sind und unter den Gefangenen häufig eine respektierte Sonderstellung genießen [41].

## Suizidprävention im Gefängnis

Wachsende Evidenz auf dem Gebiet der Suizidepidemiologie wurde Grundlage verschiedener suizidpräventiver Ansätze. Als wirksam erwiesen sich im Allgemeinen Schulungsprogramme für Ärzte und Laien, eine veränderte Berichterstattung in den Medien, die Kontrolle leicht verfügbarer Suizidmethoden, antidepressive, kognitiv-behaviorale und dialektisch-behaviorale Psychotherapie und die Nachverfolgung suizidaler Personen [42].

Da der wichtigste geschlechtsunabhängige Risikofaktor für das Versterben an Suizid das Vorliegen einer psychiatrischen Erkrankung oder einer Substanzkonsumstörung ist, hat die Identifikation individueller Risikofaktoren für die psychiatrische Arbeit besondere Bedeutung und angesichts der eingeschränkten Möglichkeiten zur Modifikation institutioneller Risikofaktoren hat die Identifizierung von Gefangenen mit erhöhtem Suizidrisiko innerhalb der Justizvollzugsanstalten ein besonderes Gewicht. Standardisierte Suizidscreeninginstrumente erfassen grundsätzlich individuelle Risikofaktoren; Effektivität und der Nutzen dieser Instrumente werden aber kontrovers diskutiert. Zum einen gibt es Evidenz dafür, dass weder individuelle Risikofaktoren noch das Vorhandensein von Suizidideen einen Gefängnissuizid valide vorhersagen können. Bei der Frage, ob der Einsatz zur Suizidprophylaxe trotzdem sinnvoll sein könnte, argumentieren Befürworter eines standardisierten Suizidscreenings, dass die Heterogenität der Untersuchungsergebnisse zum Einsatz von Suizidscreeninginstrumenten nahelegt, dass der Nutzen deutlich von der Qualifikation der Anwendenden bestimmt wird [43].

Trotz eines Mangels an aussagekräftigen Studien zum Einsatz von Suizidscreeninginstrumenten im Gefängnis, insbesondere auch von Studien mit prospektivem Design [44], wird der Einsatz von Suizidscreeninginstrumenten als Maßnahme zur Suizidprävention in Justizvollzugsanstalten empfohlen [45]. Da alle Gefangenen bei Aufnahme in den Justizvollzug ärztlich untersucht werden und ein Gespräch mit der zuständigen Fachkraft für soziale Arbeit führen, kann dieses Screening innerhalb der Routineabläufe der Justizvollzugsanstalten ressourcenneutral umgesetzt werden.

Von 8 klinisch erprobten standardisierten Suizidscreeninginstrumenten, die für die Anwendung im Gefängnis entwickelt worden sind, wurden 2, der in Österreich entwickelte „VISCI“ (Viennese Instrument for Suicidality in Correctional Institutions) und das in Berlin entwickelte Instrument „SIRAS“ (Scale for Initial Risk ASessment) von Dahle, Lohner und Konrad, das aus dem übersetzten und modifizierten niederländischen Instrument von Blaauw et al. [46] entstanden ist, als gut geeignet identifiziert [47]. Für die Entwicklung des VISCI wurden in Österreich alle Gefängnissuizide im Zeitraum von 1977 bis 1999 mit einem retrospektiven Fallkontrolldesign untersucht und auch suizidpräventive Ansätze integriert [48].

Die Untersuchung der Wirksamkeit suizidpräventiver Maßnahmen im Gefängnis ist dadurch erschwert, dass trotz der erhöhten Suizidraten von Gefangenen der Tod durch Suizid im Gefängnis insgesamt ein seltenes Ereignis ist. In einer Berliner Untersuchung wurde 2018 in der Untersuchungshaftanstalt für Männer als bedarfsorientierte Maßnahme die standardisierte Erfassung der Suizidalität mit dem SIRAS-Suizidscreening eingeführt. Eine Interventionsgruppe, in der die Suizidgefährdung mit dem SIRAS erfasst worden war, wurde mit einer Gruppe verglichen, bei der die Suizidgefährdung intuitiv-klinisch vorgenommen wurde. Dieses Design war als ein geeigneter Ansatz für die Beantwortung der Fragestellung zur Wirksamkeit aufzufassen, da ein Prognoseinstrument direkt einem klinischen Management gegenübergestellt wurde. Die Ergebnisse konnten zeigen, dass die angebotenen Maßnahmen, beispielsweise psychologische Gespräche, durch das vorangegangene Screening gezielt den Personen zugutekam, die nach Ergebnis des Screenings als stark gefährdet einzuschätzen waren, ohne dass die Zahl der Maßnahmen insgesamt zugenommen hatte [49].

Die Wirksamkeit des SIRAS-Suizidscreenings sollte auch bei Strafgefangenen, Gefangenen, die eine Ersatzfreiheitsstrafe verbüßen, jungen Gefangenen und weiblichen Gefangenen untersucht werden. Trotz des geringen zeitlichen Aufwands des SIRAS wurde dieser nach dem Ergebnis einer ergänzenden Befragung von den Untersuchenden als belastend empfunden [50]. Dieses Ergebnis unterstreicht die Notwendigkeit, bei der intramuralen Suizidprävention die Identifikation und Behandlung von Hochrisikopersonen durch Schulungen des Gefängnispersonals zu ergänzen.

## Fazit

Zur Verbesserung der Gefängnissuizidprävention sollten bei der Planung der medizinischen und psychiatrischen Versorgung die besonderen Bedürfnisse von Untersuchungsgefangenen, von weiblichen und von älteren Gefangenen berücksichtigt werden. Standardisiertes Suizidscreening sollte Teil der Aufnahmeroutine im Gefängnis sein. Bei künftigen Untersuchungen von Gefängnissuiziden sollte die Auswertung medizinischer Daten eingeschlossen werden, um die Häufigkeit von psychiatrischen Erkrankungen und Substanzkonsumstörungen verlässlich zu erfassen. Für die fachlich fundierte Begleitung suizidal gefährdeter Gefangener sollten folgende Maßnahmen kombiniert werden:

1. antidepressive Behandlung in Form stützender Gespräche, je nach Ausprägung der Symptomatik durch die Verordnung einer sedierenden, antidepressiven oder antipsychotischen Medikation,
2. Vermittlung einer sinnvollen Tagesstruktur, beispielsweise durch Aufnahme einer Arbeitstätigkeit,
3. Nachverfolgung initial suizidal gefährdeter Personen, um bei einer krisenhaften Verschlechterung rechtzeitig eingreifen zu können.
